# Psychological distress, burnout, and coping strategies among Nigerian primary school teachers: a school-based cross-sectional study

**DOI:** 10.1186/s12889-021-12397-x

**Published:** 2021-12-30

**Authors:** Eyuche Lawretta Ozoemena, Olaoluwa Samson Agbaje, Levi Ogundu, Amaka Harry Ononuju, Prince Christian Iheanachor Umoke, Cylia Nkechi Iweama, George Usman Kato, Augustina Chikaodili Isabu, Akor John Obute

**Affiliations:** 1 Department of Human Kinetics and Health Education, Faculty of Education, University of Nigeria, Nsukka, 4100001 Nigeria; 2 Department of Nursing Sciences, Faculty of Health Sciences, Bingham University, Karu, Nassarawa State Nigeria; 3 Faculty of Nursing, Niger-Delta University, Wilberforce Island, Bayelsa State Nigeria

**Keywords:** Psychological stress, Burnout, Coping strategies, Teachers, Nigeria

## Abstract

**Background:**

The teaching profession is highly stressed job. A high level of stress is associated with poor health outcomes, such as burnout and psychological distress. Therefore, teachers’ use of coping styles becomes imperative. However, relatively little is known about primary school teachers’ psychological distress, burnout, coping strategies, and associated factors in Nigeria. The study investigated psychological distress, burnout, coping strategies among primary schools, and associated factors in Nigeria.

**Methods:**

A total of 264 teachers aged 20–59 years participated in the study between May 2019 to October 2019. Questionnaires on psychological distress, burnout, coping strategies, and demographic profile form were used for data collection. Factors associated with psychological distress, burnout, and coping strategies were identified using *t*-test, univariate ANOVA, Pearson’s correlation, Chi-square test, and hierarchical linear regression analysis.

**Results:**

Of 264 participants enrolled for the study, 253 responded, giving a response rate of 95.8%. The prevalence of psychological distress and burnout was 69.9% (176/253) and 36.0% (91/253), respectively. Sex (β = 0.158), a high level of emotional exhaustion (β = 0.193) and reduced personal accomplishment (β = 0.358), adoption of problem-focused strategies (β = 0.904), and dysfunctional strategies (β = 0.340) were positively associated with psychological distress. Age (β = − 0.338), academic qualification (β = − 0.210), and income level (β = − 0.146) were inversely associated with psychological distress, which together explained 51.5% of the total variance. Psychological distress (β = 0.275 vs. β = 0.404) was significantly associated with emotional exhaustion (EE) and reduced personal accomplishment (PA) and explained 11.4 and 24.2% of the variance in EE and reduced PA, respectively.

**Conclusions:**

The high prevalence of psychological distress and burnout among teachers should receive urgent attention. Teachers’ training curricula should include developing interpersonal skills, stress management abilities, and resilience to equip them for the job. Also, teacher training curricula should integrate mental health promotion interventions.

**Supplementary Information:**

The online version contains supplementary material available at 10.1186/s12889-021-12397-x.

## Background

Teachers experience excessive occupational stress in the school environment. The teaching profession is highly stressful [1, 2], and it is associated with poor health outcomes [2, 3]. Examples of such poor health outcomes include psychological distress and burnout. Also, primary school teachers experience higher psychological distress levels than other workers from the general population [4]. Previous studies suggested that psychological distress (PD), which manifests in the form of anxiety, sadness, irritability, self-consciousness, and emotional vulnerability, is strongly associated with physical illness, diminished quality of life, reduced life span, increased uptake of health services [5], restlessness, sadness, worthlessness, nervousness, hopelessness, and helplessness [6].

Furthermore, psychological distress has been linked to burnout [7]. Burnout refers to a prolonged response to chronic physical, emotional, and mental exhaustion at work, which is characterized by emotional exhaustion (EE), depersonalization (DEP), and reduced personal accomplishment (PA) or professional efficacy (PE) [7, 8]. Maslach et al. [9] described burnout as “a psychological syndrome in response to chronic interpersonal stressors on the job, p. 398.” The definition could suggest that burnout results from prolonged exposure to work-related stressors. Burnout is associated with absenteeism, intention to leave the job, actual turnover, lower productivity, and poor job performance [7, 8]. Health-related outcomes include the involvement of physical illness and mental problems, musculoskeletal pain, cardiovascular diseases, depression, and anxiety [1, 7–12].

A systematic review and meta-analysis [13] of many occupational settings showed that risk factors such as high demands, low job control, high workload, low reward, and job insecurity increased the odds for workers’ burnout symptoms [1]. Examples of tasks or events that contribute to the teachers’ heavy workloads of include administrative paperwork (marking and grading of students’ scripts), time pressure [14], participation in meetings and workshops, managing children’s disruptive behaviors or misbehaviors, constant adjusting and re-adjusting to new pedagogical approaches, especially in the digital era, poor psychosocial work conditions (i.e., poor relationships with supervisor, co-workers, pupils/students), lack of job autonomy, role ambiguity, poor school climate, and limited resources [15–19].

Previous studies [20–24] have identified a link between mental ill-health, psychological stress, and maladaptive or dysfunctional coping strategies among workers in diverse occupational settings. Empirical research supports coping strategies as plausible mechanisms for handling stressful situations [20, 22]. Lazarus and Folkman [25] described coping as “constantly changing cognitive and behavioral efforts to manage specific external and/or internal demands that are taxing or exceeding the person’s resources, p.141.” Further, coping strategies are employed when the demands surpass individual resources. Nevertheless, coping may mitigate or worsen the adverse psychological health outcomes [26] and stimulate protective or harmful impacts on individuals’ health and wellbeing. Thus, the impact of any coping strategy is dependent on the type employed by the individuals [27, 28].

Coping strategies have been categorized into problem-versus emotion-focused, functional versus dysfunctional, approach versus avoidance, engagement versus disengagement, and primary versus secondary control coping [26, 29, 30]. However, Lazarus and Folkman’s [25] categorization of coping behaviors into problem-focused or emotion-focused seems to be the most widely used typology in studies that explored coping strategies. Problem-focused coping involves the individual’s proactive actions to effectively manage stressful situations and promptly tackle or eliminate the stressors. Contrarily, emotion-focused coping is an effort to manage emotions attributed to stressful situations without changing the situation itself. Examples of emotion-focused coping behaviors include distancing, self-controlling, accepting responsibility, escape/avoidance, and positive reappraisal [25]. Besides Lazarus and Folkman’s [25] categorization, there is also a consensus in extant literature that another dimension exists [31]. The categorization includes emotion-focused strategies (acceptance, emotional, social support, humor, positive reframing, and religion), problem-focused strategies (active coping, instrumental support, and planning), and dysfunctional coping strategies (behavioral disengagement, denial, self-distraction, self-blaming, substance use, and venting) [31, 32]. Teachers can employ coping strategies for the mitigation of mental health problems. However, many teachers adopt maladaptive or dysfunctional coping strategies with attendant health consequences, reduced productivity, and poor performance [33, 34].

Health behavior models are valuable for understanding the relationship between work stress and health outcomes among workers in diverse populations. The transactional model of stress (TMS) model has been used extensively in explaining work-related stress, burnout, and coping strategies [25, 35]. The TMS highlights the dynamic psychological interface between the stressor and the individual (i.e., person-environment interactions). The process of interactions between the stressor spans two stages: primary and secondary appraisals. At the primary appraisal, the person recognizes or perceives the prevailing situation or event (stressor) as a threat, neutral, or positive challenge. During the secondary appraisal, the person decides how to expend available resources to cope effectively. However, the stages may vary substantially among individuals [25].

Besides personality characteristics [35–37], research evidence suggests that personal characteristics influence teachers’ psychological distress, burnout, and coping strategies [38, 39]. For example, factors such as gender, education, marital status, and work experience have been identified to influence workers’ psychological distress and burnout experience [38–41]. Previous studies [40, 42, 43] reported gender differences in psychological stress, burnout, and coping among representative samples. However, the results are mixed regarding the impacts of personal characteristics on psychological distress and burnout among workers, including teachers [39–44]. Such variations could also exist in teachers’ psychological distress, burnout, and coping strategies in developing countries such as Nigeria.

Studies [45, 46] reported that teachers operate in environments with the highest risk of stress-related disorders in Nigeria. For instance, Okwaraji and Aguwa [45] reported that the prevalence of psychological distress was 32.9% among secondary school teachers in Enugu city. Also, the prevalence of burnout was 40% for emotional exhaustion, 39.4% for depersonalization, and 36.8% for reduced personal accomplishment. Also, primary school teachers in Nigeria face heavy workloads, children’s aggressive behaviors or misbehavior, poor psychosocial work conditions, role ambiguity, poor school climate, limited resources, and high numbers of pupils in one class.

From a prevention viewpoint, understanding the prevalence of psychological distress, burnout, and coping strategies and factors associated with increased risk for these outcomes among teachers is incredibly important. This is crucial to examine among teachers, as identification of risk factors for the development of poor mental outcomes can aid in better risk assessment as well as targeted development of prevention and intervention programs in the teaching profession, thereby possibly mitigating risk for adverse health outcomes among the teachers and their pupils. However, little is known about the prevalence of psychological distress, burnout, coping strategies, and associated factors among primary school teachers in southeast Nigeria. Hence, the present study has three aims: (1) to measure the prevalence of psychological distress and burnout among a sample of primary school teachers; (2) to determine the coping strategies for psychological distress and burnout in teachers; and (3) to identify the socio-demographic factors associated with psychological distress, burnout, and coping strategies among the teachers. Such an inquiry may be a critical research priority in the teaching profession in Nigeria.

## Methods

### Study setting and design

The study was conducted in Nsukka Local Government Area (LGA), Enugu North Senatorial District, Enugu State in Southeast Nigeria. Southeast Nigeria is the indigenous homeland of the Igbo people. It is one of the six geopolitical zones in Nigeria. The size of the region is 41,440 km^2^. Also, it consists of five federating units/states: Abia, Anambra, Ebonyi, Enugu, and Imo states. The study was conducted in Nsukka Local Government Area (LGA from May 2019 to October 2019 in public primary schools. Nsukka LGA has 14 autonomous communities. Some of the communities are designated in urban and rural areas. The population of Nsukka LGA, according to the National Population Commission figures, was 160,030 [47]. There are three development centers in Nsukka LGA, namely Nsukka Central, Nsukka East, and Nsukka West. The inhabitants consist of predominantly Igbo ethnic origin. However, there are people from different ethnic groups in Nigeria. There are 44 public primary schools in Nsukka central LGA [48]. Furthermore, there were 941 primary school teachers across the primary schools during the 2018/2019 academic session [48]. Private schools were exempted from the study due to the non-approval status of some private schools. The school-based cross-sectional study design was employed.

#### Inclusion and exclusion criteria

The inclusion criteria included being a teacher employed by the Enugu State Civil Service Commission, currently teaching in a public primary school in Nsukka LGA, the absence of ill health, and the issuance of voluntary informed consent. Exclusion criteria included refusal to participate in the study and ill-health.

#### Sample size and sampling procedure

A priori sample size estimation and post hoc power analysis were conducted using G*Power version 3.1 software [49, 50]. A priori sample size estimation for the intended multiple regression analysis with six independent variables (predictors), a desired statistical power of 0.95, an expected effect size of 0.26, and a significance level of 5% (0.05) produced a sample size of 238 subjects (Additional file 1). Furthermore, 10% (0.1) of the required minimum sample size was added for non-response rate (238 × 0.1 = 26). Thus, the final sample for the study was 264. For the post hoc analyses, the final sample size (i.e., 264), the number of predictors (independent variables), the significance level (0.05), and the effect size achieved (0.29) were used, respectively (Additional file 2). The results showed that the minimum sample size of 264 was adequate for the study.

### Sampling procedure

Multi-stage sampling was used to select study participants. First, we randomly selected five urban and five rural communities in Nsukka LGA. The sampling frame (primary schools) containing lists of the teachers was obtained from the Nsukka Local Government Education Authority. The primary schools were selected using simple random sampling. We contacted Headteachers at the selected schools and informed them about the study before conducting interviews. Subsequently, the teachers were selected by convenience sampling until the study’s intended sample size was reached. After completing the questionnaires, filled copies were returned to the principal investigators. We obtained verbal informed consent from the teachers who agreed to participate voluntarily. Subsequently, we administered self-report questionnaires to 264 participants. Interviews were conducted face-to-face, and each interview lasted, on average, 30–45 min.

#### Informed consent

The participants who were willing to participate in the study gave verbal informed consent before completing the questionnaires. Respondents completed the questionnaires in their offices/classrooms. All the study procedures and the administered questionnaires were fully compliant with the Declaration of Helsinki, and the University of Nigeria, Nsukka Institutional Review Board (IRB) approved the study’s protocol (Ref. Number: NHREC/05/01/2008B-FWA00002458-IRB00002323) before the study was conducted. The IRB permitted the participants to issue verbal informed consent before enrollment in the study.

### Measures

The socio-demographic characteristics of the participants were measured using a profile form developed by the investigators. The items comprise information on age, sex, academic qualification, marital status, income level, and residence place. The outcome variables of psychological distress, burnout, and coping behaviors were measured using the 12-item General Health Questionnaire (GHQ-12), the Maslach Burnout Inventory-General Survey (MBI-GS), and the Brief COPE.

### Demographic characteristics of the participants

The present study participants responded to a questionnaire that included items on socio-demographic characteristics (e.g., age, sex, educational qualification, marital status, income level, and place of residence). We coded the participants’ age in years as a continuous and discrete variable. Participants’ age was categorized as 20–29 years coded as 1; 30–39 years coded as 2, and ≥ 40 years coded as 3. Sex was coded as male (1) and female (2). Educational qualification was grouped into three categories: Ordinary National Diploma/National Certificate of Examination-OND/NCE (coded as 1), first degree-B.Sc., B.Ed., and B.A. (coded as 2), having master’s degree-M.Sc., M.A., M.Ed. (coded as 3). Marital status was categorized into four groups, single (coded 1), married (coded as 2), divorced/separated (coded as 3), and widowed (coded as 4). Income level was grouped into three categories as follows: #18,000.00 - #49,000/$36 -$98 (coded as 1), #50,000.00 -#99,000.00/$100 - $198 (coded as 2), and ≥ #100,000.00/≥ $200 (coded as 3). The place of residence was categorized into the urban area (coded as 1) and rural area (coded as 2).

### Outcome variables

#### Psychological distress

Psychological distress was measured by the 12-item General Health Questionnaire (GHQ-12). The GHQ-12 was developed as a screening instrument for identifying persons likely to have mental problems and may require health care [51]. The GHQ-12 has two different scoring procedures. The GHQ binary scoring method (0,0,1,1) and the Likert-type scoring method, where a value of 0, 1, 2, or 3, is assigned to each response format and summed across all 12 items to produce a continuous distribution of the total score ranging from 0 to 36. In the present study, both scoring procedures were employed. The GHQ binary scoring method (0,0,1,1) was designed to identify individuals reporting adequate psychological. The total number indicates that their psychological state is worse than usual is added, giving a scale score ranging from 0 to 12. The total score is used to classify the case, and higher scores indicate psychological distress. In this study, we used a cut-point of ≥3 to classify the participants. In other words, subjects scoring adverse on 3 or more of the 12 items were considered probable cases of minor psychiatric disorder. The use of the scoring procedures is consistent with recommendations for use in the general population [51–54]. The GHQ-12 has been used in previous Nigerian studies [53, 54]. The Cronbach’s alpha coefficient for the GHQ-12 was .60 (Additional file 3).

### Burnout

Burnout was measured using the 16-item version of the Maslach Burnout Inventory-General Survey (MBI-GS) [55, 56]. The MBI-GS items cover three dimensions: emotional exhaustion (EE) (5 items), depersonalization (DE) or cynicism (CY) (5 items), and personal accomplishment/professional efficacy (PE) (6 items). Examples of items in the MBI-GS include ‘I feel emotionally drained from my work (exhaustion)’, ‘I doubt the significance of my work (depersonalization/cynicism)’; and ‘I am good at my job (personal efficacy).’ All items are scored on a 7-point rating scale ranging from 0 (never) to 6 (daily/every day). As recommended [55, 56], high scores on EE and CY/DE and low scores on PA indicate burnout (i.e., all PA-items are reversibly scored). The MBI and MBI-GS have been used in Nigerian studies [57, 58]. The teachers were considered to experience burnout if they scored ≥24 points for EE or ≥ 9 points for CY/DE or < 19 points for PA [56, 59, 60]. Accordingly, we used the cut-off points on EE and DE scales to classify the participants into those “*with burnout*” or “*without burnout*.” The Cronbach’s alpha coefficients for EE, CY, and PA subscales were .53, .52, and .94 (Additional files 4). The permission to use the MBI-GS for this study was received from Mind Garden, Inc.

### Coping strategies

The Brief Coping Orientation for Problem Experiences (COPE) was used to collect information about teachers’ responses. The instrument has 28 items that measure 14 factors of 2 items each. The scale is assigned a 4-point Likert scale ranging from 0 “I have not been doing this at all (0)”, “I have been doing this a little bit (1)”, to “I have been doing this a lot (3)”. The Brief COPE has 14 subscales (self-distraction, emotional support, instrumental support, active coping, denial, substance use, positive reframing, planning, behavioral disengagement, venting, humor, acceptance, self-blame, and religion) consist of two subscales each. We categorized the strategies into emotion-focused strategies, problem-focused strategies, and dysfunctional coping strategies [32]. We categorized participants based on their approval or use of coping strategies if they reported at least “*I have been doing this a little bit*.” We also report the percentages of the participants who employed each coping strategy. The Cronbach’s alpha coefficients for the emotion-focused coping, problem-focused coping, and dysfunctional coping subscales were .74, .78, and .73, respectively (Additional file 5).

### Data quality management

Before data collection, data collectors and supervisors received 1 day of training from the principal investigator. A pre-test was conducted in Nsukka East LGA, and 20 teachers were selected for the procedure. No revision was conducted on the instruments because the items in the GHQ-12, MBI-GS, and Brief COPE are relevant to the work environment contexts in Nigeria. The teachers did not complain about their comprehension and appropriateness of the instruments in measuring the outcomes. Data were checked for completeness during data collection.

### Statistical analysis

Descriptive analyses were performed on the demographic data to describe the sample, frequencies, percentages, mean, and standard deviations. We conducted normality tests to assess the outcome variables’ distribution using the Kolmogorov-Smirnov test, and the data distribution was normal. We considered a normal distribution when skewness and kurtosis were within the range [− 2, 2]. Pearson’s correlation analysis was computed to examine the relationship between the study variables. Distributions of the MBI-GS, GHQ-12 and Brief COPE scores in the demographics were tested by the Independent Samples *t*-test and one-way analysis of variance (ANOVA). The Eta squared (*η*^2^) values were computed to determine the effect size. We used Cohen’s *d* recommendations to interpret the effect size values [61]. Thus, there is a small (*d* = 0.2), medium (*d* = 0.5), and large (*d* = 0.8) effect size. The possible multicollinearity between the independent variables was evaluated using the variance inflation factor (VIF < 10). Hierarchical linear regression analyses examined the relationships between demographic factors, psychological distress, burnout, and coping strategies. The standardized estimate (β), *F*, *R*^2^, and *R*^2^-changes (Δ*R*^2^) for each step were calculated. All data analyses were performed using SPSS version 25 software for Windows IBM Corp., Armonk, NY, USA). The significance level was established a priori at *p* < 0.05 (two-tailed).

## Results

### Socio-demographic characteristics of the participants

Of 264 participants enrolled in the study, 253 responded, giving a response rate of 95.8% (mean age 33.1 ± 8.93 years). More than half were females (65.2%), and about half were graduates (46.6%). Most of the participants were married (78.3%). Also, most of the participants were low-income earners (i.e., #18, 000.00 - #49,000.00) (48.6%), and majority lived in urban areas (76.6%). Other demographic information was presented in Table 1.

**Table 1:** Prevalence of psychological distress, burnout, and coping strategies by participants’ characteristics (*N* = 253)

Participant’s characteristics	*n* (%)	Prevalence of PD	Prevalence of burnout	Work-related coping strategies		
		GHQ-12 ≥ 3	EE ≥ 24 + DP ≥ 9	^c^EFS	^d^PFS	^e^DCS
		*n* (%)	*n* (%)	*n* (%)	*n* (%)	*n* (%)
	All sample	176 (69.6)	91 (36.0)	139 (54.9)	107 (42.3)	194 (76.7)
**Age (Years)**						
20–29 years	58 (22.9)	51 (87.9)	1 (1.7)	53 (91.4)	25 (43.1)	48 (82.8)
30–39 years	62 (24.5)	47 (75.8)	41 (66.1)	42 (67.7)	22 (35.5)	42 (67.7)
≥ 40 years	133 (52.6)	78 (58.6)	49 (36.8)	114 (85.7)	60 (45.1)	104 (76.7)
χ^2^		17.780***	54.064***	13.537**	1.627	4.139
**Sex**						
Male	88 (34.8)	59 (67.0)	33 (37.5)	70 (79.5)	39 (44.3)	68 (77.3)
Female	165 (65.2)	117 (70.9)	58 (35.2)	139 (84.2)	68 (41.2)	126 (76.4)
χ^2^		0.405	0.137	0.881	0.227	0.027
**Academic qualification**						
OND/NCE	72 (28.5)	55 (76.4)	26 (36.1)	67 (93.1)	30 (41.7)	67 (93.1)
B.Sc./B.Ed./B.A.	118 (46.6)	88 (74.6)	41 (34.7)	89 (75.4)	48 (40.7)	74 (62.7)
^a^Master’s degree	63 (24.9)	33 (52.4)	24 (38.1)	53 (84.1)	29 (46.0)	53 (84.1)
χ^2^		11.770**	0.201	9.811**	0.499	25.626***
**Marital status**						
Single	30 (11.9)	25 (83.3)	20 (66.7)	20 (66.7)	11 (36.7)	20 (66.7)
Married	198 (78.3)	136 (68.7)	61 (30.8)	164 (82.8)	81 (40.9)	154 (77.8)
Widow/divorced/separated	25 (9.9)	15 (60.0)	10 (40.0)	25 (100.0)	15 (60.0)	20 (80.0)
χ^2^		3.839	14.741**	10.577**	3.756	1.970
**Monthly Income**						
^b^#18, 000.00 - #49,000.00	123 (48.6)	91 (74.0)	45 (36.6)	98 (79.7)	36 (29.3)	93 (75.6)
#50, 000.00 - #99,000.00	40 (15.8)	31 (77.5)	21 (52.5)	31 (77.5)	10 (25.0)	21 (52.5)
≥ #100, 000.00	90 (35.6)	54 (60.0)	25 (27.8)	80 (88.9)	61 (67.8)	80 (88.9)
χ^2^		6.213*	7.388*	3.934**	37.401***	20.659***
**Place of residence**						
Urban	194 (76.7)	134 (69.1)	64 (33.0)	153 (78.9)	91 (46.9)	138 (71.1)
Rural	59 (23.3)	42 (71.2)	27 (45.8)	56 (94.9)	16 (27.1)	56 (94.9)
χ^2^		0.096	3.205	8.111**	7.259**	14.308***

*Note.* PD, psychological distress; OND, Ordinary National Diploma; NCE, National Certificate of Examination; B.Sc., Bachelor of Science; B.Ed., Bachelor of Education; B.A., Bachelor of Arts; Master’s degree, Master of Science (M.Sc.); Master of Education (M.Ed.); Master of Arts (M.A.); EFS, emotion-focused strategies; PFS, problem-focused strategies; DCS, dysfunctional coping strategies; ^b^#18, 000, this was the national minimum wage at the time of the study. The new national minimum wage is #30,000 per month for civil servants including teachers; ^c^EFS, only the proportion of participants that used EFS; ^d^PFS, only the proportion of participants that used PFS; ^e^DCS, only the proportion of participants that used DCS

* *p* < .05; ** *p* < .001; ***p < .0001

### Prevalence of psychological distress and burnout

The prevalence of psychological distress and burnout was 69.9% (176/253) and 36.0% (91/253), respectively. The prevalence of burnout was 15.8% (40/253) for EE, 26.1% (66/253) for DE and 84.6% (214/253) for diminished/reduced PA (Table 3). In addition, there were statistically significant difference in the prevalence of psychological distress among participants in groups by age (χ^2^ (251) = 17.780, *p* < 0.0001), academic qualification (χ^2^ (251) = 11.770, *p* = 0.003), and monthly income (χ^2^ (251) = 6.213, *p* = 0.045). Also, statistical significance differences were found in the proportion of participants with burnout by age (χ^2^ (251) = 54.064, *p* < 0.0001), marital status (χ^2^ (251) = 14.741, *p* = 0.001), and monthly income (χ^2^ (251) = 7.388, *p* = 0.025) (Table 1).

### Coping strategies of teachers

The majority of our sample (76.7%) adopted dysfunctional strategies. Also, 54.9 and 42.3% used emotion-focused and problem-focused coping styles. There were also statistically significant differences in the proportion of participants who employed specific coping strategies in groups by age, academic qualification, income, and residence (Table 1).

### Psychological distress, burnout, and coping strategies of teachers

The total mean psychological distress score was 16.80 ± 4.86. Therefore, the majority of the teachers had a psychiatric disorder. The total mean burnout score was 46.60 ± 15.76. The teachers reported the highest score in the PA subscale (27.3 ± 9.65) of the MBI-GS, followed by EE and DE subscales. The total mean scores for the EE (13.04 ± 7.96) and DE (6.29 ± 6.06) subscales showed that teachers had a moderate burnout level. The Brief COPE score of coping strategies was 34.32 ± 18.23. Based on the adopted categorization of coping strategies [31, 32], the results show that teachers adopted more of the dysfunctional strategies (24.30 ± 8.73) and emotion-focused (22.71 ± 8.43) than the problem-focused strategies (14.05 ± 5.89). Furthermore, there were statistically significant differences in psychological distress between participants in groups by age (*F* = 12.887, *p* < 0.0001), gender (*t* = − 2.047, *p* = 0.042) and academic qualification (*F* = 6.535, *p* = 0.002). There were also statistically significant differences in burnout between participants in groups by age (*F* = 11.393, *p* < 0.0001), academic qualification (*F* = 5.147, *p* < 0.0001), marital status (*F* = 8.335, *p* < 0.0001), and monthly income (*F* = 6.649, *p* = 0.002). Furthermore, statistically significant differences were found in the coping strategies between participants in groups by age (*F* = 6.974, *p* = 0.001), academic qualification (*F* = 7.528, *p* = 0.001), monthly income (*F* = 11.502, *p* < 0.0001), and place of residence (*t* = − 2.112, *p* = 0.037). The Eta squared shows that effect size ranges from small to large (*η*^2^ = .0004–.13). Table 2 contains other significant results.

**Table 2:** Comparison of psychological distress, burnout, and coping strategies by participants’ characteristics (*N* = 253)

Participant’s characteristics	Mean (SD)/*n* (%)	GHQ-12 total score (M ± SD)	MBI-GS total score (M ± SD)	MBI-GS dimensions			Brief-COPE total score (M ± SD)
				EE	DE	PA/PE	
				(M ± SD)	(M ± SD)	(M ± SD)	
**Age (Years)**	33.1 (8.93)						
20–29 years	58 (22.9)	18.9 ± 3.41	38.6 ± 10.93	9.6 ± 5.11	2.7 ± 3.22	26.3 ± 9.82	40.6 ± 14.75
30–39 years	62 (24.5)	17.6 ± 4.73	51.1 ± 15.61	16.8 ± 8.84	8.8 ± 7.10	25.5 ± 9.65	28.4 ± 18.92
≥ 40 years	133 (52.6)	15.5 ± 5.05	47.9 ± 16.45	12.8 ± 7.96	6.7 ± 5.78	28.5 ± 9.48	34.4 ± 18.47
*F*		12.887***	11.393***	13.639***	17.908***	2.484	6.974**
*η*^2^		.09	.08	.10	.13	.02	.05
**Sex**							
Male	88 (34.8)	15.9 ± 5.57	44.4 ± 18.27	12.6 ± 8.08	7.1 ± 7.09	24.7 ± 11.59	34.4 ± 19.12
Female	165 (65.2)	17.2 ± 4.39	47.8 ± 14.16	13.3 ± 7.90	5.9 ± 5.40	28.6 ± 8.15	34.3 ± 17.79
*t*		−2.047*	−1.657	−0.704	1.566	−3.150**	0.076
*η*^2^		.01	.01	.001	.01	.04	
**Academic qualification**							
OND/NCE	72 (28.5)	18.4 ± 3.56	45.0 ± 15.79	11.7 ± 7.67	7.1 ± 7.85	26.2 ± 10.17	40.9 ± 12.72
B.Sc./B.Ed./B.A.	118 (46.6)	16.6 ± 5.91	44.7 ± 17.92	12.5 ± 8.45	6.6 ± 5.20	25.6 ± 10.47	30.6 ± 21.10
^a^Master’s degree	63 (24.9)	15.5 ± 3.29	52.0 ± 8.84	15.6 ± 6.76	4.8 ± 4.94	31.6 ± 5.32	33.8 ± 15.74
*F*		6.535**	5.147**	4.831**	2.631	8.836***	7.528**
*η*^2^		.05	.04	.04	.02	.07	.06
**Marital status**							
Single	30 (11.9)	16.5 ± 2.66	52.3 ± 7.72	17.5 ± 5.11	9.4 ± 7.42	25.4 ± 10.46	30.4 ± 21.52
Married	198 (78.3)	16.8 ± 5.32	44.6 ± 16.46	11.9 ± 8.02	5.7 ± 5.59	26.9 ± 9.80	34.6 ± 18.35
Widow/divorced/separated	25 (9.9)	16.8 ± 2.61	55.8 ± 12.20	16.8 ± 7.40	7.2 ± 6.04	31.8 ± 5.57	36.6 ± 11.72
*F*		0.078	8.335***	10.276***	5.461**	3.466*	0.931
*η*^2^		.001	.06	.08	.04	.03	.007
**Monthly Income**							
^b^#18, 000.00 - #49,000.00	123 (48.6)	17.5 ± 5.59	43.0 ± 18.09	11.6 ± 8.92	5.9 ± 5.70	25.6 ± 10.36	32.1 ± 17.47
#50, 000.00 - #99,000.00	40 (15.8)	15.9 ± 5.60	51.4 ± 18.73	13.7 ± 6.96	10.6 ± 5.55	27.2 ± 9.92	26.4 ± 15.40
≥ #100, 000.00	90 (35.6)	16.2 ± 2.98	49.3 ± 8.09	14.7 ± 6.58	4.9 ± 5.97	29.7 ± 7.98	40.9 ± 18.41
*F*		2.926	6.649**	4.150*	13.576***	4.867**	11.502***
*η*^2^		.02	.05	.03	.10	.04	.08
**Place of residence**							
Urban	194 (76.7)	16.8 ± 5.40	47.6 ± 16.15	13.7 ± 7.78	5.9 ± 5.76	28.0 ± 9.41	33.1 ± 18.94
Rural	59 (23.3)	16.9 ± 2.37	43.5 ± 14.10	10.9 ± 8.25	7.8 ± 6.80	24.8 ± 10.10	38.2 ± 15.14
*t*		−0.248	1.740	2.324*	−2.126*	2.272*	−2.112*
*η*^2^		.0004	.01	.02	.02	.02	.02

*Note.* PD, psychological distress; EE, emotional exhaustion; DE, depersonalization; PE/PA, personal self-efficacy/personal accomplishment; OND, Ordinary National Diploma; NCE, National Certificate of Examination; B.Sc., Bachelor of Science; B.Ed., Bachelor of Education; B.A., Bachelor of Arts; Master’s degree, Master of Science (M.Sc.); Master of Education (M.Ed.); Master of Arts (M.A.); ^b^#18, 000, this was the national minimum wage at the time of the study. The new national minimum wage is #30,000 per month for civil servants, including teachers

* *p* < .05; ** *p* < .001; ***p < .0001

### Correlation among psychological distress, burnout, and work-related coping strategies

The total mean psychological distress score was 16.80 ± 4.86. The result shows a high level of psychological distress among the teachers. Pearson correlation analysis indicated that psychological distress was positively correlated with burnout and two of the three subscales-EE and PA (*r* = 0.215; 0.358, *p* < 0.001), and positively associated with coping strategies and the three subscales (*r* = 0.439–0.356, *p* < 0.001). The total mean burnout score was 46.6 ± 15.76. This is indicative of a moderate level of burnout among primary school teachers. Burnout was positively correlated with coping strategies (*r* = 0.261, p < 0.001) and its subscales of emotion-focused, problem-focused, and dysfunctional strategies (*r* = 0.339–0.340, *p* < 0.001). The Brief COPE scores (*r* = 0.376, p < 0.001) and its three subscales were all positively correlated with psychological distress (*r* = 0.439–0.356, *p* < 0.05). Other correlations are presented in Table 3.

**Table 3:** Descriptive statistics and intercorrelations among study variables

Variables	*M*	*SD*	1	2	3	4	5	6	7	8	9
Psychol. distress (GHQ-12 score)	16.8	4.86	–								
Burnout (MBI-GS score)	46.6	15.76	0.33**	–							
Emotional Exhaustion (EE)	13.0	7.96	0.22**	0.76**	–						
Depersonalization (DE)	6.30	6.06	0.01	0.52**	0.36**	–					
Personal accomplishment (PA)	27.3	9.65	0.45**	0.68**	0.19**	−0.08	–				
Coping (Brief COPE score)	34.3	18.23	0.48**	0.27**	0.21**	−0.14**	0.51**	–			
Emotion-focused strategies	22.7	8.43	0.44**	0.34**	0.12	−0.09	0.52**	0.95**	–		
Problem-focused strategies	14.1	5.60	0.51**	0.33**	0.19**	−0.17**	0.49**	0.86**	0.82**	–	
Dysfunctional strategies	24.3	8.73	0.36**	0.34**	0.26**	−0.11	0.41**	0.92**	0.78**	0.66**	–

*Note. M*, mean; *SD*, standard deviation; GHQ-12, 12-item General Health Questionnaire; MBI-GS, Maslach Burnout Inventory-General Survey, Psychol., psychological; Brief COPE, Brief Coping Orientation for Problem Experiences

*p < .05; **p < .001

### Factors associated with psychological distress and burnout

In the hierarchical linear regression analyses presented in Table 4, participants’ age, sex, academic qualification, marital status, and monthly income were entered into step 1 to act as control variables, since these factors were significantly related to psychological distress as indicated by the *t*-test and univariate ANOVA (Table 2). The dimensions of burnout (EE, DE, and PA) were entered into Step 2 as control variables. In step 3, total coping scores and scores on emotion-focused, problem-focused, and dysfunctional strategies were entered into the model. Psychological distress was used as the dependent variable. In Step 1, age (β = − 0.338) and gender (β = 0.158) were significantly associated with psychological distress and accounted for 14.0% of the variance in PD. In Step 2, after adjusting for the demographic variables, age (β = − 0.301), academic qualification (β = − 0.210), a high level of EE (β = 0.193) and reduced personal accomplishment (β = 0.358) were significantly associated with PD. These variables together explained 31.1% of the variance. In the third step, age (β = − 0.172), academic qualification (β = − 0.171), and income level (β = − 0.146) were inversely associated with PD. Also, gender (β = 0.142), decreased personal accomplishment (β = 0.138), adoption of problem-focused strategies (β = 0.904), and dysfunctional strategies (β = 0.340) were significant predictors of PD. These variables altogether explained 51.5% of the total variance (Table 4).

**Table 4:** Hierarchical linear regression analyses of factors associated with psychological distress (GHQ-12 scores**)**

Variables	Psychological distress		
	Step 1 (*β*)	Step 2 (*β*) †	Step 3 (*β*)
Age	−0.338***	− 0.301***	−0.172*
Sex	0.158*	0.094	0.142**
Academic qualification	−0.163	−0.210*	− 0.171*
Marital status	0.090	0.041	−0.102
Income level	0.145	0.068	−0.146*
Emotional exhaustion (EE)		0.193**	0.097
Depersonalization (DE)		0.012	0.019
Personal accomplishment (PA)		0.358***	0.138*
Coping (Brief COPE scores)			−0.941***
Emotion-focused strategies			0.203
Problem-focused strategies			0.904***
Dysfunctional strategies			0.340**
*R*^2^	0.140	0.311	0.515
Adjusted *R*^2^	0.119	0.289	0.489
∆*R*^2^	0.140	0.171	0.204
*F*	6.647***	12.171***	19.516***

*Note.* B, beta; *F,* F-distribution; *R*^2^ variance; GHQ-12, 12-item version of the General Health Questionnaire; Brief COPE, Brief Coping Orientation for Problem Experiences; Step 2 (β)† adjusted for age, sex, academic qualification, marital status, income level and place of residence

* *p* < .05; ** *p* < .001; ***p < .0001

In Table 5, participants’ age, sex, academic qualification, marital status, monthly income, and residence place were entered into step 1 to act as control variables since these factors were significantly related to burnout as indicated by the *t*-test and univariate ANOVA (Table 2). Psychological distress was entered into step 2. In step 3, total coping scores and scores on emotion-focused, problem-focused, and dysfunctional strategies were entered into the model. The EE, DE, and PA were used as dependent variables. The demographic variables were not significantly associated with EE for this first step. In Step 2, after adjusting for the demographic variables, psychological distress (β = 0.275) was significantly associated with EE and explained 11.4% of the variance in EE. In the third step, place of residence and coping were significantly reversely associated with EE. However, the use of dysfunctional strategies by teachers positively predicted EE. The model explained 23.9% of the variance in EE.

**Table 5:** Hierarchical linear regression analyses of factors associated with burnout (MBI-GS scores**)**

Variables	Emotional exhaustion			Depersonalization			Personal Accomplishment		
	Step 1 (*β*)	Step 2(*β*)†	Step 3 (*β*)	Step 1 (*β*)	Step 2 (*β*)	Step 3 (*β*)	Step 1 (*β*)	Step 2 (*β*)	Step 3 (*β*)
Age	−0.044	0.049	0.093	0.522***	0.556***	0.527***	−0.096	0.040	0.119
Sex	0.027	−0.017	0.022	−0.048	−0.064	− 0.058	0.167**	0.103	0.110*
Academic qualif.	0.095	0.140	0.152	−0.321***	−0.305***	− 0.358***	0.090	0.156	0.358***
Marital status	0.009	−0.016	−0.005	− 0.145*	−0.154*	− 0.153*	0.135*	0.099	0.037
Income level	.0119	0.079	−0.054	−0.112	− 0.127	−0.010	0.156	0.097	−0.212**
Place of residence	−0.098	−0.085	− 0.205**	0.091	0.095	0.111	−0.075	− 0.057	−0.153**
Psychol. distress		0.275***	0.158*		0.100	0.090		0.404***	0.146*
Brief COPE scores			−0.753**			−1.309***			−0.583**
EFS			0.014			0.709***			0.760***
PFS			0.182			0.107			0.060
DFS			0.833**			0.366**			0.397**
*R*^2^	0.049	0.114	0.239	0.179	0.188	0.327	0.102	0.242	0.467
Adjusted *R*^2^	0.026	0.089	0.204	0.159	0.165	0.296	0.080	0.221	0.442
∆*R*^2^	0.049	0.065	0.125	0.179	0.009	0.139	0.102	0.140	0.224
*F*	2.123	4.517***	6.876***	8.950***	8.092***	10.650***	4.660***	11.201***	19.162***

Note. *F* F-distribution; *R*^2^ variance; qualif. Qualification; Psychol. Psychological; Brief COPE, Brief Coping Orientation for Problem Experiences; EFS, Emotion-focused strategies; PFS, Problem-focused strategies; DFS, Dysfunctional strategies

Step 2 (*β*)† adjusted for age, sex, academic qualification, marital status, income level and place of residence

* *p* < .05; ** *p* < .001; ***p < .0001

For depersonalization, age, sex, academic qualification, marital status, monthly income, and residence place were entered into step 1 to act as control variables. Age was significantly associated with DE. However, academic qualification and marital status were significantly and inversely associated with DE. The model explained 17.9% of the variance in DE. In step 2, age was also significantly associated with DE.

Similarly, academic qualification and marital status were significantly reversely associated with DE. The variables explained 18.8% of the variance in DE. Age, EFS, and DFS were positively associated with DE in the third step. However, academic qualification and marital status were inversely related to DE. These factors explained 32.7% of the variance in DE.

For personal accomplishment, the demographic factors were entered into step 1 to act as control variables. Sex and marital status were significantly associated with reduced PA, which explained 10.2% of the variance in PA. In step 2, psychological distress was entered into the model. Psychological was a significant positive predictor of low levels of PA and explained 40.4% of the variance in reduced PA. In Step 3, the total coping scores and forms of coping strategies were entered into the model. Sex, academic qualification, psychological distress, EFS, and DFS were significantly associated with decreased PA. Psychological was a significant positive predictor of reduced PA and explained 14.6% of the variance in PA. However, income level (β = − 0.212), place of residence (β = − 0.153), and total coping (β = − 0.583) were significantly and reversely correlated with PA. The model explained 46.7% of the variance in PA.

## Discussion

### Principal findings

This study investigated the prevalence of psychological distress, burnout, coping strategies, and their associations with demographic factors among primary school teachers in southeast Nigeria. To our knowledge, this is the first study evaluating psychological distress and burnout, the use of coping strategies, and their associations with demographics. The results showed that the overall prevalence of psychological distress and burnout was 69.9% (176 of 253 teachers) and 36.0% (91 of 253 teachers). Therefore, a high proportion of primary school teachers suffered from mental health symptoms. The prevalence of psychological distress was higher than the reported prevalence in previous studies [4, 20, 45].

In the school environment, primary school teachers are overwhelmed with meeting the pupils’ learning, social needs, and some cases, health needs. The teachers’ inability to fulfill these needs may lead to psychological distress. Also, a perceived lack of control or uncertainty over pupils’ misbehaviors or dysfunctional behaviors may create depressive and anxiety symptoms in teachers [5, 6]. The unavailability of instructional resources in resource-constrained settings, limited incentives, and a lack of administrative and parental support, which characterized Nigerian primary education settings are also significant sources of distress in primary school teachers. In this study, these factors plausibly may explain the high proportion of teachers with psychological distress.

The prevalence of burnout was 15.8% (40/253) for EE, 26.1% (66/253) for DE, and 84.6% (214/253) for diminished PA. Similarly, the prevalence of burnout was higher than the reported prevalence in previous studies [41]. However, the prevalence of EE and DE was similar to a previous study [34] and lowered compared with another previous study [45]. We assume that scarce teaching and learning resources, poor school climate, excessive workloads, exposure to adverse events (e.g., verbal aggression and bullying), and poor remunerations of Nigerian teachers might be plausible reasons to explain why the teachers essentially experienced both psychological distress and burnout. Reducing teachers’ occupational stress and improving the schools’ working conditions might significantly decrease psychological distress and risk of teachers’ burnout and improve their overall health [41]. Our findings may confirm the underlying assumptions of transactional models of stress (TMS) in that differences in teachers’ attributes within the schools in perceptions of demands of situation and resources of the person’s psychological, biological, or social systems are risk factors for psychological distress and burnout symptoms [25].

In this study, a substantial majority (76.7%) of teachers adopted dysfunctional strategies. Only 42.3% used problem-focused coping styles. The finding is in line with a prior study [20]. Evidence-based interventions focusing on mental health and problem-focused coping behaviors are needed for Nigerian teachers. Besides, demographic characteristics of age, academic qualification, income, and place of residence were significantly associated with teachers’ use of specific coping strategies, which is consistent with a previous study [22].

Furthermore, there were statistically significant differences in psychological distress among participants in groups by sex, age, and academic qualification. Female teachers experienced psychological distress more than male teachers, which was consistent with a previous study [43]. Females generally face more pressures than males, and many females experience both work-and family life-related stressors daily [43]. For instance, females perform domestic activities, including childcare and caring for the sick. Thus, the persistent exposure to pressures at home and work triggers work-family life conflicts and may make female teachers more susceptible to psychological distress. The finding is consistent with previous studies [2, 22, 43, 45]. Interventions that promote female teachers’ mental health outcomes are expedient.

Teachers aged between 20 and 29 years old experienced a higher level of psychological distress. Teachers in this age group are mostly inexperienced and struggle to cope with the demands of the teaching profession, such as managing children’s disruptive behaviors or misbehavior, continually adjusting and re-adjusting to new pedagogical approaches, poor school climate, and limited resources [15–19]. Aging may not necessarily provide a buffer for work stressors. However, as workers age, they acquire requisite skills, job-related experiences, and work conditions to improve resilience to stressors. This could explain the lower psychological distress levels observed in older teachers. The finding is consistent with previous studies [2, 22, 45]. Hence, improved in-service training, and better support at the beginning of a working career, and responsive practices may help nurture improved adaptation to the teaching profession.

There were also statistically significant differences in burnout between participants by age, academic qualification, marital status, and monthly income. In our study, older teachers experienced burnout more than younger teachers. The plausible explanation for the finding could be that older teachers feel tired and exhausted by the enormous responsibilities. Additionally, the poor working conditions and meager resources in many Nigerian elementary schools may explain this finding, as proposed by the transactional stress model [25, 35]. Thus, workplace interventions could target social support and better psychosocial work conditions in Nigerian elementary schools, especially older teachers. However, older teachers had a higher level of PA than younger teachers. This probably might be attributed to the perceived sense of fulfillment over many years in the teaching profession. The finding is consistent with a previous study [45]. However, the finding contradicts previous studies [2, 62]. The discrepancies in findings could be due to prevailing working conditions, teachers’ resources, and school climate in China and Canada compared to Nigeria.

Our study further showed that teachers’ burnout symptoms differed significantly by academic qualification. Teachers with a master’s degree had a higher level of burnout than teachers with NCE/OND and B.Sc. degrees. The rational explanation for the outcome could be that teachers with higher educational qualifications were assigned more complex tasks than the less qualified teachers. Also, teachers with lower educational qualifications such as OND/NCE may be ill-prepared to manage the teaching profession’s pressures due to the quality of training and job-related experience acquired. Thus, they may require the professional support of teachers with higher academic qualifications. These situations could make the job of teachers with higher qualifications more demanding. The finding contradicts a previous study [45]. Thus, teachers with higher educational qualifications need more job-related resources to minimize the risk of burnout.

The widowed/separated/divorced teachers experienced higher burnout than married and single teachers, which agreed with previous studies [2]. Literature suggests that married individuals receive social support from their partners, which plays a significant role in coping with job stressors. Hence, married teachers are less vulnerable to physical and psychological strains [2]. Further, this category of teachers experienced high burnout due to the burden associated with widowhood, grief associated with the loss/death of a spouse, or loneliness. The loss of a spouse has been linked to poor mental health outcomes, especially among women. Hence, combined with stressful working conditions, the widowed/separated/divorced teachers’ high burnout levels could be reasonable. Therefore, mental health interventions are needed by this category of teachers to alleviate their burnout experience.

Teachers with higher income levels had a higher burnout experience than teachers with low-income levels. The finding is inconsistent with a previous study [2]. Furthermore, higher-income teachers had more EE than low-income earners. However, high-income earners had higher PA than low-income earners. The finding may suggest that teachers’ income levels may significantly mediate their burnout symptoms. Nevertheless, this claim requires longitudinal studies to ascertain its veracity.

The hierarchical linear regression analysis further showed that age and sex were significantly associated with psychological distress. This supports previous studies’ findings that demographic factors are significant predictors of psychological distress [2, 22, 45]. However, age and sex explained only 14.0% of the variance in PD. Also, the relatively small value of adjusted *R*^2^ of age and sex implies that other contributing factors that were not included in the study can explain differences in the teachers’ psychological distress. After adjusting for the demographic variables, age, academic qualification, a high EE level, and perceived decreased personal accomplishment were associated with PD. This supports the findings of previous studies [2, 22, 40, 45]. These variables combined to explain 31.1% of the variance in PD. Besides the demographic variables, a low level of personal accomplishment, adoption of problem-focused strategies, and dysfunctional strategies were significant predictors of PD [40]. These variables altogether explained 51.5% of the total variance in PD. Thus, future studies could explore if the observed relationship is causal via longitudinal studies.

Also, after adjusting for the demographic variables, psychological distress was significantly associated with EE. The finding is consistent with previous studies [2, 21, 41, 45]. However, psychological distress only explained 11.4% of the variance in EE. Similarly, the relatively low value of adjusted *R*^2^ of psychological distress suggests that other contributing factors not included in the study can explain differences in the teachers’ burnout. Teachers’ place of residence, psychological distress, and use of dysfunctional strategies positively predicted EE. The finding confirms that psychological distress [2, 45] and dysfunctional or maladaptive strategies of self-blame, venting, denial, substance use, behavioral disengagement, self-distraction, and positive reframing [20] contribute to EE. The model explained 23.9% of the variance in EE. The finding emphasizes the need to modify contextual factors (school climate and culture) and job-related factors (excessive workloads and a dearth of resources) in Nigerian primary schools. The teachers also need social support from colleagues (i.e., peer support) and leadership support (i.e., headmistresses/headteachers/supervisors).

Age, academic qualification, and marital status were significantly associated with DE. This supports the findings of previous studies [2, 22, 45]. The demographic factors altogether explained 17.9 and 18.8% of the variance in DE in steps 1 and 2, respectively. The EFS and DFS also were significantly associated with DE. This supports the findings of previous studies [2, 22, 45]. The demographic factors combined with EFS and DFS explained 32.7% of the variance in DE. Using large samples further studies could explore the combined effects of several demographic factors on DE.

Additionally, our results showed that sex and marital status were significantly associated with reduced PA and explained 10.2% of the variance in PA. Also, psychological distress was a positive predictor of reduced PA and explained 24.2% of the variance. Also, sex, academic qualification, psychological distress, EFS, and DFS were significantly associated with decreased PA. However, income level, place of residence, and total coping were significantly and inversely related to reduced PA. These explained 46.7% of the variance in PA. Consistent with previous studies [2, 8, 22], females, especially married females, experience work-family life conflicts, leading to job burnout and impeding personal accomplishments in chosen careers. Besides, they also experience domestic violence, workplace sexual harassment, or bullying in Nigeria [63, 64]. These events combined to impact women’s mental health; therefore, personal accomplishments are significantly undermined [63, 64]. Also, psychological distress was a positive predictor of reduced PA and explained 24.2% of the variance in PA. The finding is consistent with previous studies [41, 45].

Academic qualification plays a vital role in teachers’ accomplishments. Also, having higher education levels may potentially facilitate higher PA among teachers. However, low academic qualification could limit teachers’ professional achievements. Therefore, it becomes imperative for the teachers’ training curriculum in Nigeria to integrate content that enhances professional and practical skills, fostering psychosocial competence and professional skills. Previous studies [17, 20, 65] had emphasized the considerable impact of teachers’ interpersonal skills and stress management abilities. Possession of these skills could improve teachers’ PA regardless of academic qualification. The use of emotion-focused strategies (EFS) and dysfunctional strategies (DFS) was significantly associated with decreased PA in our sample. The findings agreed with previous studies [2, 45]. Interventions that promote the adoption of adaptive strategies among teachers are needed.

However, income level, residence, and total coping were significantly and inversely related to reduced PA. These explained 46.7% of the variance in PA. Due to Nigeria’s high poverty level, we assume that teachers with higher income levels may perceive themselves as successful because they can afford more personal or professional development resources. Such factors could improve their professional development and competence. The urban-rural differences could significantly impact teachers’ PA in Nigeria. Teachers in the urban areas have access to infrastructures and opportunities (e.g., universities, colleges of education, job opportunities, etc.) that could enhance PA compared to those in the rural areas. Thus, urban teachers have more capacities and resources to improve their professional achievements than rural teachers. The finding suggests that rural teachers require more professional development opportunities and material resources to promote their PA. Future studies could explore the impacts of residence types on teachers’ accomplishments.

### Limitations

This study has several notable limitations. Due to the cross-sectional study design, the causal relations between psychological distress, burnout, and demographic characteristics could not be examined. Future longitudinal studies are needed to confirm the current findings of the study. Also, biases-response, recall, and selection could be introduced into the study. One of the major limitations of this study is the use of cut-off scores for the dimensions of burnout. According to the MBI authors, this approach does not have diagnostic validity, and its use is discouraged. Future studies should adopt the procedures for data analysis as recommended by the MBI authors. Self-report measures could introduce social desirability and may not adequately represent the teachers’ psychological and burnout symptoms. Future efforts can combine a mixed-method approach to understand the study findings better. Also, all participating teachers were in public primary schools; findings cannot be generalized to secondary and private schools. Therefore, extrapolations must be made with caution. Future studies should use large samples that include private and public schools’ teachers.

### Implications for practice

As a result of a high proportion of teachers who reported psychological distress and burnout and their association with dysfunctional coping strategies, implementing multi-components evidence-based mental health interventions would be valuable at the individual and organizational levels to improve teachers’ mental health and support the teachers’ effective performance of duties. Our findings have practical implications for health education, promotion interventions, and occupational health. Moreover, since more teachers employed dysfunctional coping strategies, future interventions to increase teachers’ usage of problem-focused or adaptive coping strategies are needed. Teachers’ training curriculum should also integrate professional competence, development, and resilience to cope effectively with their professional demands.

## Conclusions

This study found that many teachers suffered from psychological distress and a high proportion of teachers had burnout symptoms. Our findings showed that psychological distress predicted burnout and its dimensions among the teachers. Also, some demographic factors were associated with psychological distress and burnout. Preventive measures and mental health interventions to reduce teachers’ psychological distress and burnout may be critical to preventing psychiatric disorders among Nigeria teachers. Our study further showed that a high proportion of teachers used dysfunctional strategies. Therefore, effective stress preventive measures and problem-focused strategies are recommended, particularly among female and beginning teachers. Consequently, teachers’ training curricula should include developing interpersonal skills and stress management abilities to equip them for the job.

## Supplementary Information

**Additional file 1.**

**Additional file 2.**

**Additional file 3.**

**Additional file 4.**

**Additional file 5.**

**Additional file 6.**

**Additional file 7.**

## Data Availability

All the data generated or analyzed during the study are included in this published article. The raw data of this research can be obtained from contacting the authors.

## Abbreviations

PD: psychological distress
OND: Ordinary National Diploma
NCE: National Certificate of Examination
B.Sc.: Bachelor of Science
B.Ed.: Bachelor of Education
B.A.: Bachelor of Arts
M.Sc.: Master of Science
M.Ed.: Master of Education
M.A.: Master of Arts
EFS: emotion-focused strategies
PFS: problem-focused strategies
DCS: dysfunctional coping strategies
LGA: Local government area
AOR: adjusted odds ratio
CI: confidence interval
OR: odds ratio
VIF: variance inflation factor
MBI-GS: Maslach Burnout Inventory-General Survey
GHQ-12: the 12-item version of General Health Questionnaire
COPE: Coping Orientation for Problem Experiences
