# Supplementary material for: Psychological distress, burnout, and coping strategies among Nigerian primary school teachers: a school-based cross-sectional study
Source: BMC Public Health. 2021 Dec 30;21:2327. doi: 10.1186/s12889-021-12397-x (PMC8719383; doi:10.1186/s12889-021-12397-x)
Supplement: Supplementary file 1 — Additional file 1. [file 12889_2021_12397_MOESM1_ESM.rtf]

Additional file 1: A priori: compute required sample size-given á, power, and effect size 


[1] -- Monday, May 27, 2019 -- 17:00:10
Exact - Linear multiple regression: Random model
Options:	Exact distribution
Analysis:	A priori: Compute required sample size 
Input:	Tail(s)	=	Two
	H1 ñ²	=	0.2653907
	H0 ñ²	=	0.10
	á err prob	=	0.05
	Power (1-â err prob)	=	0.95
	Number of predictors	=	6
Output:	Lower critical R²	=	0.0551348
	Upper critical R²	=	0.2028229
	Total sample size	=	238
	Actual power	=	0.9503140
