# Supplementary material for: Psychological distress, burnout, and coping strategies among Nigerian primary school teachers: a school-based cross-sectional study
Source: BMC Public Health. 2021 Dec 30;21:2327. doi: 10.1186/s12889-021-12397-x (PMC8719383; doi:10.1186/s12889-021-12397-x)
Supplement: Supplementary file 2 — Additional file 2. [file 12889_2021_12397_MOESM2_ESM.docx]

[1] *-- Tuesday, August 25, 2020 -- 18:06:22*

**Exact -** Linear multiple regression: Random model

**Options:** Exact distribution

**Analysis:** Post hoc: Compute achieved power

**Input:** Tail(s) = Two

H1 ρ² = 0.2868173

H0 ρ² = 0.10

α err prob = 0.05

Total sample size = 262

Number of predictors = 6

**Output:** Lower critical R² = 0.0561150

Upper critical R² = 0.1966109

Power (1-β err prob) = 0.9887415
