# Supplementary material for: Psychological distress, burnout, and coping strategies among Nigerian primary school teachers: a school-based cross-sectional study
Source: BMC Public Health. 2021 Dec 30;21:2327. doi: 10.1186/s12889-021-12397-x (PMC8719383; doi:10.1186/s12889-021-12397-x)
Supplement: Supplementary file 6 — Additional file 6. [file 12889_2021_12397_MOESM6_ESM.docx]

**Additional File 6.** STROBE Checklist

|  | **Item no** |  |
| --- | --- | --- |
| **Title and Abstract** | 1 | Psychological distress, burnout, and coping strategies among Nigerian primary school teachers: A school-based cross-sectional study  Title page, P1, Line 1-2.  Abstract, P2, Line 31-56 |
| **Introduction** |  |  |
| Background/rationale | 2 | In Nigeria, studies [46-47] reported that teachers operate in environments with the highest risk of stress-related disorders. For instance, Okwaraji and Aguwa [46] reported that the prevalence of psychological distress was 32.9% among secondary school teachers in Enugu city. Also, the prevalence of burnout was 40% for emotional exhaustion, 39.4% for depersonalization, and 36.8% for reduced personal accomplishment. Also, primary school teachers in Nigeria face heavy workloads, children's aggressive behaviors or misbehavior, poor psychosocial work conditions, role ambiguity, poor school climate, limited resources, and high numbers of pupils in one class. However, little is known about the prevalence of psychological distress, burnout, coping strategies and associated factors among primary school teachers in southeast Nigeria.  Background, paragraph 8-9, P5, Line 131-142 |
| Objectives | 3 | The present study has three aims: (1) to measure the prevalence of psychological distress and burnout among a sample of primary school teachers; (2) to determine the coping strategies for psychological distress and burnout in teachers, and (3) to identify the sociodemographic factors associated with psychological distress, burnout, and coping strategies among the teachers. Such an inquiry may be a critical research priority in the teaching profession in Nigeria.  Background, Paragraph 1, P6, Line 145-150. |
| **Methods** |  | P6, Line 151 |
| Study design | 4 | Institutional based Cross-sectional study. Methods, Paragraph 1, P6, Line 162 |
| Setting | 5 | The study was conducted in Nsukka LGA, Enugu State. Nsukka LGA is one of the LGAs in Enugu State. The study period covered five months from May to October, 2019.  Methods, Paragraph 1, P6, Line 153-162. |
| Participants | 6 | The population for the study comprised 941 primary school teachers across the primary schools during the 2018/2019 academic session [49].  Methods, Paragraph 1, P6, Line 159-161.  The inclusion criteria included being a teacher employed by the Enugu State Civil Service Commission, and currently teaching in a public primary school in Nsukka LGA, the absence of ill health, and issuance of voluntary informed consent. Exclusion criteria included refusal to participate in the study and ill-health.  Methods, Paragraph 2, P6, Line 164-167. |
| Variables | 7 | The primary outcome variables were psychological distress, burnout, and coping strategies. Methods (measures), Paragraph 1, P7-10, Line 196-258. |
| Data sources/  measurement | 8 | Psychological distress  Psychological distress was measured by the 12-item General Health Questionnaire (GHQ-12). The GHQ-12 was developed as a screening instrument for identifying persons likely to have mental problems and may require health care [52].  Burnout  Burnout was measured using the 16-item version of the Maslach Burnout Inventory-General Survey (MBI-GS) [56-57]. The MBI-GS items cover three dimensions: emotional exhaustion (EE) (5 items), depersonalization (DE) or cynicism (CY) (5 items), and personal accomplishment or professional efficacy (PE) (6 items).  Coping strategies  The Brief Coping Orientation for Problem Experiences (COPE) was used for the collection of information about teachers' responses. The instrument has 28 items that measure 14 factors of 2 items each. The scale is assigned a 4-point Likert scale ranged from 0 "I have not been doing this at all (0)", “I have been doing this a little bit (1)”, to "I have been doing this a lot (3)". The Brief COPE has 14 subscales (self-distraction, use of emotional support, instrumental support, active coping, denial, substance use, positive reframing, planning, behavioral disengagement, venting, humor, acceptance, self-blame, and religion) consist of two subscales each.  Methods (measures), Paragraph 3, P8-12, Line 215-258. |
| Bias | 9 | To address recall bias, the participants were asked to self-report their psychological distress, burnout and coping strategies in the past 12 months. In addition, to address information bias, we used well-validated instruments for data collection.  Methods (measures), Paragraph 3, P8-12, Line 215-258. |
| Study size | 10 | A priori sample size estimation and post hoc power analysis were conducted using G*Power version 3.1 software [50-51]. A priori sample size estimation for the intended multiple regression analysis with six independent variables (predictors), a desired statistical power of 0.95, an expected effect size of 0.26, and a significance level of 5% (0.05) produced a sample size of 238 subjects (Additional file 1). Furthermore, 10% (0.1) of the required minimum sample size was added for non-response rate (238 x 0.1 = 26). Thus, the final sample for the study was 264. For the post hoc analyses, the final sample size (i.e., 264), the number of predictors (independent variables), the significance level (0.05), and the effect size achieved (0.29) were used, respectively (Additional file 2). The results showed that the minimum sample size of 264 was adequate for the study.  Methods (Sample size and procedure),  Paragraph 3, P6-7, Line 168-177. |
| Quantitative variables | 11 | To assess teachers’ psychological distress, the GHQ binary scoring method (0,0,1,1) was designed to identify individuals reporting adequate psychological. The total number of times a person indicates that their psychological state is worse than usual is added, giving a scale score ranging from 0 to 12. The total score is used for classification of the case, and higher scores indicate psychological distress. In this study, we used a cut-point of ≥ 3 to classify the participants. In other words, subjects scoring adverse on 3 or more of the 12 items were considered probable cases of minor psychiatric disorder. The use of the scoring procedures is consistent with recommendations for use in the general population [52-55].  Methods (Measures), Paragraph 3, P9, Line 223-229.  To assess burnout, we used the recommended [56-57], scoring protocol; high scores on EE and CY/DE and low scores on PA are indicative for burnout (i.e., all PA-items are reversibly scored). The MBI and MBI-GS have been used in Nigerian studies [58-59]. The teachers were considered to experience burnout if they scored ≥ 24 points for EE or ≥ 9 points for CY/DE or < 19 points for PA [57, 60-61]. Accordingly, we used the cut-off points on EE and DE scales to classify the participants into those “with burnout” or “without burnout."  Methods (Measures), Paragraph 4, P9, Line 239-244.  To measure use of coping strategies, we categorized participants based on their approval or use of coping strategies if they reported at least “I have been doing this a little bit." We also reported the percentages of the participants who employed each coping strategy.  Methods (Measures), Paragraph 4, P10, Line 254-257. |
| Statistical methods | 12 | Descriptive analyses were performed on the demographic data to describe the sample, frequencies, percentages, mean, and standard deviations. We conducted normality tests to assess the outcome variables' distribution using the Kolmogorov-Smirnov test, and the data distribution was normal. We considered a normal distribution when skewness and kurtosis were within the range [-2, 2]. Pearson’s correlation analysis was computed to examine the relationship between the study variables. Distributions of the MBI-GS, GHQ-12, and Brief COPE scores in the demographics were tested by the Independent Samples t-test and one-way analysis of variance (ANOVA). The Eta squared (η2) values were computed to determine the effect size. We used Cohen’s d recommendations for the interpretation of the effect size values [62]. Thus, there is a small (0.01 ≤ d ≤ 0.05), moderate (0.06 ≤ d ≤ 0.13), or large (d ≥ 0.14) effect size. The possible presence of multicollinearity between the independent variables was evaluated using the variance inflation factor (VIF < 10). Hierarchical linear regression analyses were used to examine the relationships between demographic factors, psychological distress, burnout, and coping strategies. The standardized estimate (β), F, R2, and R2-changes (ΔR2) for each step were calculated. All data analyses were performed using SPSS version 25 software for Windows IBM Corp., Armonk, NY, USA). The significance level was established a priori at p < 0.05 (two-tailed).  Methods (statistical analysis), Paragraph 1, P10, Line 264-279. |
| **Results** |  | P11, Line 282 |
| Participants | 13 | Of 264 participants enrolled for the study, 253 responded, giving a response rate of 95.8% (mean age 33.1 ± 8.93 years). More than half were females (65.2%), and about half were graduates (46.6%). Most of the participants were married (78.3%). Also, most of the participants were low-income earners (i.e., #18, 000.00 - #49,000.00) (48.6%), and majority lived in urban areas (76.6%). Other demographic information was presented in Table 1.  Results section, Paragraph 1, P11, Line 282-288. |
| Descriptive data | 14 | The prevalence of burnout was 15.8% (40/253) for EE, 26.1% (66/253) for DE and 84.6% (214/253) for diminished/reduced PA (Table 3). In addition, there were statistically significant difference in the prevalence of psychological distress among participants in groups by age (χ2 (251) = 17.780, p < 0.0001), academic qualification (χ2 (251) = 11.770, p = 0.003), and monthly income (χ2 (251) = 6.213, p = 0.045). Also, statistical significance differences were found in the proportion of participants with burnout by age (χ2 (251) = 54.064, p < 0.0001), marital status (χ2 (251) = 14.741, p = 0.001), and monthly income (χ2 (251) = 7.388, p = 0.025) (Table 1).  Results section, Paragraph 2, P11, Line 291-297.  The total mean psychological distress score was 16.80 ± 4.86. The result shows a high level of psychological distress among the teachers. Pearson correlation analysis indicated that psychological distress was positively correlated with burnout and two of the three subscales-EE and PA (r = 0.215; 0.358, p < 0.001), and also positively associated with coping strategies and the three subscales (r = 0.439 – 0.356, p < 0.001). The total mean burnout score was 46.6 ± 15.76. This is indicative of a moderate level of burnout among primary school teachers. Burnout was positively correlated with coping strategies (*r* = 0.261, *p* < 0.001) and its subscales of emotion-focused, problem-focused, and dysfunctional strategies (*r* = 0.339 – 0.340, *p* < 0.001). The Brief COPE scores (r = 0.376, *p* < 0.001) and its three subscales were all positively correlated with psychological distress (r = 0.439–0.356, p < 0.05). Other correlations were presented in Table 3. Results section, Paragraph 5, P12, Line 322-331. |
| Outcome data | 15 | The prevalence of psychological distress and burnout was 69.9% (176/253) and 36.0% (91/253), respectively.  Results section, Paragraph 2, P11, Line 290-291.  Majority of our sample (76.7%) adopted dysfunctional strategies. Also, 54.9% and 42.3% used emotion-focused and problem-focused coping styles. There were also statistically significant differences in the proportion of participants who employed specific coping strategies in groups by age, academic qualification, income, and residence (Table 1).  Results section, Paragraph 3, P11, Line 298-302. |
| Main results | 16 | The major findings showed that participants’ age, gender, academic qualification, marital status, and monthly income were entered into step 1 to act as control variables, since these factors were significantly related to psychological distress as indicated by the *t*-test and univariate ANOVA (Table 2). The dimensions of burnout (EE, DE, and PA) were entered into Step 2 as control variables. In step 3, total coping scores and scores on emotion-focused, problem-focused, and dysfunctional strategies were entered into the model. Psychological distress was used as the dependent variable. In Step 1, age (β = -0.338) and gender (β = 0.158) were significantly associated with psychological distress and accounted for 14.0% of the variance in PD. In Step 2, after adjusting for the demographic variables, age (β = -0.301), academic qualification (β = -0.210), a high level of EE (β = 0.193) and reduced personal accomplishment (β = 0.358) were significantly associated with PD. These variables together explained 31.1% of the variance. In the third step, age (β = -0.172), academic qualification (β = -0.171), and income level (β = -0.146) were inversely associated with PD. Also, gender (β = 0.142), decreased personal accomplishment (β = 0.138), adoption of problem-focused strategies (β = 0.904), and dysfunctional strategies (β = 0.340) were significant predictors of PD. These variables altogether explained 51.5% of the total variance (Table 4).  Also, participants’ age, gender, academic qualification, marital status, monthly income, and residence place were entered into step 1 to act as control variables since these factors were significantly related to burnout as indicated by the t-test and univariate ANOVA (Table 2). Psychological distress was entered into step 2. In step 3, total coping scores and scores on emotion-focused, problem-focused, and dysfunctional strategies were entered into the model. The EE, DE, and PA were used as dependent variables. For this first step, the demographic variables were not significantly associated with EE. In Step 2, after adjusting for the demographic variables, psychological distress (β = 0.275) was significantly associated with EE and explained 11.4% of the variance in EE. In the third step, place of residence and coping were significantly reversely associated with EE. However, the use of dysfunctional strategies by teachers positively predicted EE. The model explained 23.9% of the variance in EE.  For depersonalization, age, gender, academic qualification, marital status, monthly income, and residence place were entered into step 1 to act as control variables. Age was significantly associated with DE. However, academic qualification and marital status were significantly and inversely associated with DE. The model explained 17.9% of the variance in DE. In step 2, age was also significantly associated with DE. Similarly, academic qualification and marital status were significantly reversely associated with DE. The variables explained 18.8% of the variance in DE. In the third step, age, EFS, and DFS were positively associated with DE. However, academic qualification and marital status were inversely related to DE. These factors explained 32.7% of the variance in DE.  Results section, Paragraphs 6-7, P17, Line 445-471.  For personal accomplishment, the demographic factors were entered into step 1 to act as control variables. Gender and marital status were significantly associated with reduced PA, which explained 10.2% of the variance in PA. In step 2, psychological distress was entered into the model. Psychological was a significant positive predictor of low levels of PA and explained 40.4% of the variance in reduced PA. In Step 3, the total coping scores and forms of coping strategies were entered into the model. Gender, academic qualification, psychological distress, EFS, and DFS were significantly associated with decreased PA. Psychological was a significant positive predictor of reduced PA and explained 14.6% of the variance in PA. However, income level (β = -0.212), place of residence (β = -0.153), and total coping (β = -0.583) were significantly and reversely correlated with PA. The model explained 46.7% of the variance in PA.  Results section, Paragraphs 8-9, P18, Line 472-489. |
| Other analyses | 17 | N/A |
| **Discussion** |  | Discussion section  P18, Line 491-499; P19, Line 500-527; P20, Line 528-555; P21, Line 556-583; P22, Line 584-611; P23, Line 612-639. |
| Key results | 18 | The results showed that the overall prevalence of psychological distress and burnout was 69.9% (176 of 253 teachers) and 36.0% (91 of 253 teachers). Therefore, a high proportion of primary school teachers suffered from mental health symptoms. Discussion (main findings), Paragraph 1, P18, Line 495-497.  A substantial majority (76.7%) of teachers adopted dysfunctional strategies. Only 42.3% used problem-focused coping styles.  Discussion (main findings), Paragraph 3, P19, Line 513-514. |
| Limitations | 19 | Due to the cross-sectional study design, the causal relations between psychological distress, burnout, and demographic characteristics could not be examined. Future longitudinal studies are needed to confirm the current findings of the study. Also, biases-response, recall, and selection could be introduced into the study. The use of self-report measures could introduce social desirability and may not adequately represent the teachers' psychological and burnout symptoms. Future efforts can combine a mixed-method approach to provide more understandings of the study findings. Also, because all participating teachers were in public schools, findings cannot be generalized to private schools. Therefore, extrapolations must be made with caution. Future studies should use large samples that include teachers in private and public schools.  Limitations, Paragraph 1, P24, Line 640-649. |
| Interpretation | 20 | We provided a cautious overall interpretation of our results considering objectives, limitations, multiplicity of analyses, results from similar studies, and other relevant evidence. We also considered residual confounding due to unmeasured variables such as, school climate, job demands, work-family conflicts, work conflicts, social support, and other institutional factors.  Discussion (Main findings) P18-23. |
| Generalizability | 21 | Since the data used in the current study were from a state in southeast Nigeria, and limited to only the primary school teachers, the findings may not be generalizable to all teachers who are employed in secondary schools, and private schools in Enugu State and Nigeria in general.  Limitations, Paragraph 1, Line 647-650. P27; Line 649-664, P28. |
| **Other information** |  | N/A |
| Funding | 22 | This research received no specific grant from any funding agency in public, commercial, or not-for-profit sectors. The study was funded by the authors.  Funding, P25, Line 691-692. |

*Note.* N/A = Not Applicable.
